# GLP‐1 and Dual GLP‐1/GIP Receptor Agonists in Heart Failure With Mildly Reduced or Preserved Ejection Fraction: A Systematic Review and Meta‐Analysis

**DOI:** 10.1002/clc.70234

**Published:** 2025-12-13

**Authors:** Mushood Ahmed, Muhammad Burhan, Aimen Shafiq, Tallal Mushtaq Hashmi, Raheel Ahmed, Marat Fudim, Robert J. Mentz, Gregg C. Fonarow

**Affiliations:** ^1^ Rawalpindi Medical University Rawalpindi Pakistan; ^2^ Dow University of Health Sciences Karachi Pakistan; ^3^ Department of Cardiology Royal Brompton Hospital London UK; ^4^ National Heart and Lung Institute Imperial College London London UK; ^5^ Duke University Medical Center, Division of Cardiology Durham North Carolina USA; ^6^ Duke Clinical Research Institute Durham North Carolina USA; ^7^ Ahmanson‐UCLA Cardiomyopathy Center, Division of Cardiology University of California Los Angeles Los Angeles California USA

## Abstract

This comprehensive meta‐analysis reveals that GLP‐1 and dual GLP‐1/GIP receptor agonists are associated with reduced risk of composite cardiovascular endpoints and worsening heart failure events. However, no statistically significant differences were observed regarding all‐cause or cardiovascular mortality.

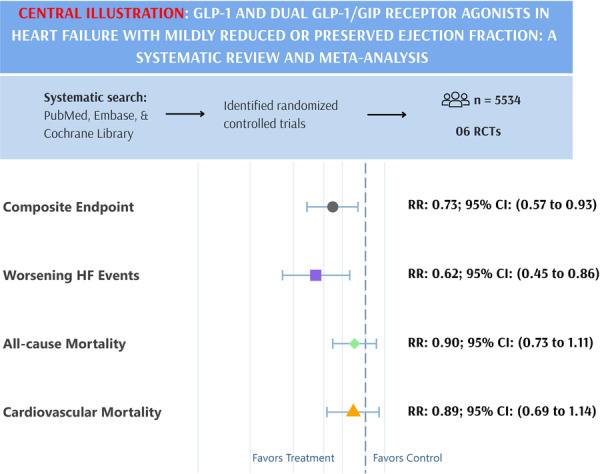

Heart failure (HF) affects more than 64 million people worldwide and causes a significant amount of morbidity, lowers the quality of life, and leads to recurrent hospitalizations [1, 2]. Among the subtypes of HF, HF with mildly reduced or preserved ejection fraction (referred as HFpEF hereafter) accounts for more than half of all HF cases globally and has proven especially challenging to treat, as its pathophysiology is often linked to metabolic disorders such as obesity, diabetes, and systemic inflammation [3, 4]. Although historically there was no conclusively effective treatment for HFpEF, over the past decade there have been tremendous advancements in its management; notably, sodium‐glucose cotransporter 2 inhibitors (SGLT‐2i), sacubitril‐valsartan and glucagon‐like peptide‐1 receptor agonists (GLP‐1 RAs). GLP‐1 RAs (including semaglutide), conceived initially as anti‐diabetic drugs for type 2 diabetes, have additional beneficial effects like body weight reduction, anti‐inflammatory properties, and modulation of cardiovascular risks [5]. These factors appear to be particularly relevant for patients with HFpEF who often present with metabolic abnormalities. Recent studies, including the SUMMIT trial, have added to the growing evidence supporting the use of tirzepatide (GLP‐1 and glucose‐dependent insulinotropic polypeptide (GIP) RA) in HFpEF as it was shown to reduce worsening HF events and improve symptom severity in patients with obesity‐related HFpEF [6]. These findings prompt a reexamination of the role of GLP‐1RA medications (such as semaglutide/exenatide) and GLP1/GIP‐RA medications (such as tirzepatide) in the management of HFpEF and their potential to complement existing therapies.

This meta‐analysis was conducted in accordance with the Cochrane Handbook for Systematic Reviews of Interventions and the Preferred Reporting Items for Systematic Reviews and Meta‐Analyses (PRISMA) guidelines [7, 8]. A comprehensive literature search was conducted across PubMed/MEDLINE, Embase, and the Cochrane Library, covering all records from the inception of each database up to November 2024 (Table S1). Studies included randomized controlled trials (RCTs) and their post hoc analyses that investigated the use of GLP‐1RA or GLP1/GIP‐RA in patients with HFpEF. The primary outcome was a composite of cardiovascular death and worsening heart failure events, while secondary outcomes included worsening HF events, all‐cause death and cardiovascular death. The definitions of composite endpoint and worsening HF events are provided in Table S2. Data analysis was performed in R version 4.4.1 using the “meta” package. Statistical heterogeneity was assessed using the I² statistic. A leave‐one‐out sensitivity analysis was conducted sequentially excluding individual studies to evaluate their impact on the overall findings for outcomes with > 40% heterogeneity.

Subgroup analysis was performed for primary outcome based on age (< 65 or ≥ 65 years, gender (male or female), BMI (< 35 or ≥ 35), systolic blood pressure (< 130 or ≥ 130) and the use of mineralocorticoid receptor antagonist. Additionally, a subgroup analysis was conducted to evaluate the pooled estimates based on the drug type (the GLP‐1RA semaglutide/exenatide *vs.* the GLP1/GIP RA tirzepatide). A *p*‐value < 0.05 was considered statistically significant for all clinical outcomes.

A total of six studies were included, all of which were RCTs or their post‐hoc analyses [9, 10, 11, 12, 13, 14]. Furthermore, we examined the participant‐level data reported by Kosiborod et al. to ensure the inclusion of relevant information from all available trials [15]. The PRISMA flowchart outlines the study selection process (Figure S1). The pooled dataset consisted of 5534 participants, with 2777 receiving intervention and 2757 in the control group. The baseline characteristics showed a well‐balanced population across intervention and control groups, with mean ages ranging from 61.7 to 69.3 years and a predominantly male demographic. Left ventricular ejection fraction (LVEF) ranged from 55% to 61%, and body mass index (BMI) varied from 34.0 to 38.3 in the intervention group and 37.2 to 38.2 in the control group. The detailed baseline characteristics, including mean age, gender distribution, BMI, dosage, and follow‐up durations, are summarized in Table 1. All trials evaluated the effects of once‐weekly subcutaneous injections of semaglutide, tirzepatide or exenatide, with follow‐up periods ranging from 12 to 40.8 months. All included trials had a low risk of bias (Figure S2). The use of GLP‐1 and GLP‐1/GIP RAs was associated with a significantly reduced risk of the composite endpoint of cardiovascular death or worsening heart failure events compared to the control group [RR: 0.73; 95% CI: 0.57 to 0.93, (*p* = 0.011) Figure 1A]. A significantly reduced risk was observed in the risk of worsening heart failure events with the intervention group [RR: 0.62; 95% CI: 0.45 to 0.86, (*p* = 0.004) Figure 1B]. No statistically significant difference was observed for all‐cause death [RR: 0.90; 95% CI: 0.73 to 1.11, (*p* = 0.320) Figure 1C] or cardiovascular death [RR: 0.89; 95% CI: 0.69 to 1.14, (*p* = 0.342) Figure 1D]. The heterogeneity of composite endpoint (I^2^ = 42.6%), and worsening heart failure (I^2^ = 43.4%) was reduced by omitting STEP‐HFpEF, 2023 (Figures S3 and S4). The subgroup analysis of primary composite endpoint based on age, gender, BMI, systolic blood pressure and use of mineralocorticoid receptor antagonist showed that no difference in the subgroups (*p*‐interaction > 0.05) (Figure 2). The subgroup analysis based on drug type did not show any significant findings (*p*‐interaction > 0.05) (Figure S5–S8).

**Table 1:** Baseline characteristics of included studies and patients.

Study, year	Sample size‐N		Mean age (SD)		Male‐N		BMI (SD)		Dose		Follow‐up (months)
	Intervention	Control	Intervention	Control	Intervention	Control	Intervention	Control	GLP‐1/GIP RA	Control	
SELECT, 2023	1174	1099	61.7 (8.7)		1566		34·0 (5·4)		Semaglutide once‐weekly S/C dose of 2.4 mg	Placebo	39.8
STEP HFpEF, 2023	263	266	69 (9.7)	68.7 (9.7)	114	118	37.4 (5.4)	37.3 (6.2)	Semaglutide once‐weekly S/C dose of 2.4 mg	S/C Placebo	12
STEP‐HFpEF DM, 2024	310	306	68.3 (8.9)	69.3 (8.9)	182	161	37.3 (5.9)	37.2 (5.7)	Semaglutide once‐weekly S/C dose of 2.4 mg	S/C Placebo	12
SUMMIT, 2024	364	367	65.5 (10.5)	65.0 (10.9)	164	174	38.3 (6.4)	38.2 (7.0)	Tirzepatide once‐weekly S/C dose of 2.5 mg	S/C Placebo	12
EXSCEL, 2024	499	561	—	—	1440	1486	—	—	Exenatide once‐weekly S/C dose of 2 mg	S/C Placebo	38.4
FLOW, 2024	167	158	—	—	—	—	—	—	Semaglutide once‐weekly S/C dose of 1.0 mg	S/C Placebo	40.8

Abbreviations: BMI, body mass index, N, number; S/C, subcutaneous.

**Figure 1:**
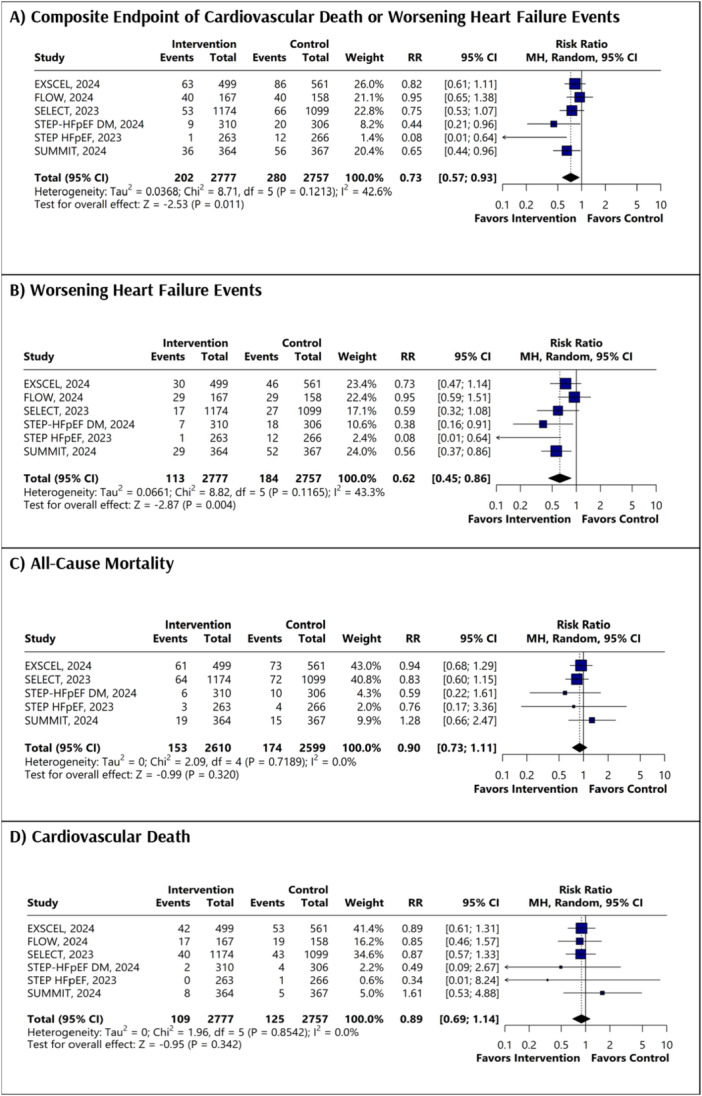
Forest plots of (A) composite endpoint, (B) worsening heart failure events, (C) all‐cause death, (D) cardiovascular death.

**Figure 2:**
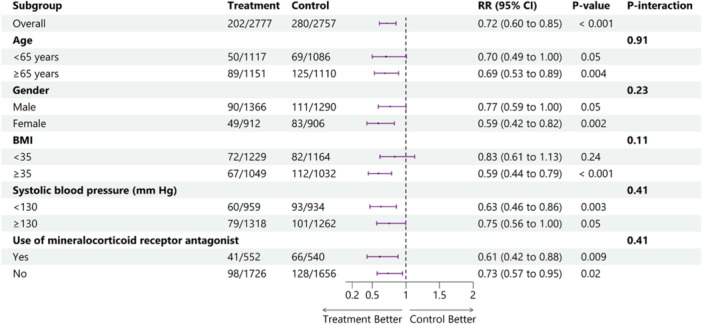
Subgroup analysis for the primary outcome based on age, sex, BMI, SBP, and use of mineralocorticoid receptor antagonists. *Note:* EXSCEL 2024 did not report the data for subgroups.

Our analysis reveals that GLP‐1 and GLP‐1/GIP RAs are associated with reduced risk of composite cardiovascular endpoints and worsening heart failure events. However, no statistically significant differences were observed regarding all‐cause or cardiovascular mortality.

Metabolic disorders such as obesity, insulin resistance, and systemic inflammation are commonly associated with HFpEF and enhance left ventricular stiffness, endothelial dysfunction, and the risk of worsening heart failure [3]. The weight‐reducing and anti‐inflammatory properties of GLP‐1 and GLP‐1/GIP RAs may add a unique therapeutic value to managing HFpEF [16]. Their role in modulating metabolic pathways offers a strong mechanistic rationale for their use in HFpEF, for which both weight loss and glycemic control are associated with improved outcomes. Notably, GLP‐1 and GLP‐1/GIP RAs may help address critical drivers of HFpEF by enhancing ventricular compliance and decreasing myocardial strain, which would slow the clinical progression of the disease.

An important finding is the reduction in worsening heart failure events observed with GLP‐1 RAs. In HFpEF patients, especially in those with comorbid metabolic conditions, such events often represent a common source of hospitalizations due to heart failure decompensation. The reduction in the incidence of such events with GLP‐1 RAs should improve quality of life and reduce overall healthcare burdens related to HFpEF. Even though treatments like SGLT2 inhibitors emerged as promising tools for HF across the EF spectrum and regardless of body weight [17], including GLP‐1 RAs in the therapeutic regimen in those individuals with obesity could offer a complementary strategy for the management of HFpEF.

The lack of significant impact on all‐cause and cardiovascular mortality requires further consideration. Mortality in HFpEF is driven by a variety of factors, including non‐cardiovascular comorbidities such as diabetes, renal dysfunction, and obesity, which might attenuate the expected survival benefits of GLP‐1 RAs. This could further indicate that longer follow‐up periods or larger populations of patients are needed to see meaningful differences in mortality. For instance, the SUMMIT trial only had a total of 34 deaths. Moreover, the heterogeneity of HFpEF itself may play a role. This suggests that the benefit of GLP‐1 RAs may be more evident in particular subsets, such as obesity‐related HFpEF or significant metabolic syndrome. Stratified analyses will determine which HFpEF subgroups can most likely benefit from GLP‐1 RA therapy.

Although GLP‐1 RAs have shown efficacy in reducing heart failure‐related events, their role among the many emerging therapies targeting HFpEF must be considered. SGLT2 inhibitors are now documented to benefit both HFpEF and HFrEF with an added benefit on rates of HF hospitalization and cardiovascular mortality [18]. Hence, GLP‐1 RAs may serve as an adjunctive therapy, primarily in those whose HFpEF is triggered by metabolic impairment. The combination of GLP‐1 RAs with SGLT2 inhibitors should be evaluated further to optimize care pathways. A recent propensity score‐matched study of 7044 patients demonstrated that patients with HFpEF who received GLP‐1 RA along with SGLT2 inhibitors had better outcomes as compared to SGLT2 inhibitors alone [19]. Despite the promising evidence for GLP‐1 RAs in HFpEF, more challenges to its broad adoption include high cost compared to some HF therapies, potentially more limited availability, and gastrointestinal side effects that could limit adherence [20]. This is the first meta‐analysis to pool the recently published SUMMIT trial to assess tirzepatide's effectiveness, while earlier studies only focused on semaglutide. We ensured our findings are robust by doing sensitivity and subgroup analyses and thoroughly reviewing all trials and post‐hoc analyses. This gives our meta‐analysis a high statistical power. However, it is important to mention that we were unable to analyze inflammatory markers, and lipid status due to considerable heterogeneity in the reporting of these characteristics across trials. Future studies should aim to examine specific subsets of HFpEF patients for whom GLP‐1 RA may offer the most benefit specifically concerning age, BMI, and other metabolic states. Further randomized studies will be important in establishing the generalizability of these treatments, across a wide variety of patients, especially their effect on all‐cause and cardiovascular death. Personalized medicine approaches that consider genetic and metabolic data might tailor GLP‐1 RA therapy and optimize its effectiveness in the treatment of HFpEF.

## Author Contributions

**Mushood Ahmed:** conceptualization, data curation, project administration, supervision, formal analysis, methodology, software, writing – original draft. **Muhammad Burhan:** formal analysis, methodology, software, writing – original draft. **Aimen Shafiq:** writing – original draft. **Tallal Mushtaq Hashmi:** formal analysis, methodology, software. **Raheel Ahmed:** writing –reviewing and editing. **Marat Fudim:** visualization, validation, writing – reviewing, and editing. **Robert J. Mentz:** visualization, validation, writing – reviewing and editing. **Gregg C. Fonarow:** conceptualization, data curation, project administration, visualization, validation, writing – reviewing, and editing.

## Conflicts of Interest

Gregg C. Fonarow reported receiving personal fees from Abbott, Amgen, AstraZeneca, Bayer, Boehringer Ingelheim, Cytokinetics, Eli Lilly, Johnson & Johnson, Medtronic, Merck, Novartis, and Pfizer outside the submitted work. Marat Fudim reported receiving personal fees from Alleviant, Ajax, Alio Health, Alleviant, Artha, Audicor, Axon Therapies, Bayer, Bodyguide, Bodyport, Boston Scientific, Broadview, Cadence, Cardioflow, Cardionomics, Coridea, CVRx, Daxor, Deerfield Catalyst, Edwards LifeSciences, Echosens, EKO, Feldschuh Foundation, Fire1, FutureCardia, Galvani, Gradient, Hatteras, HemodynamiQ, Impulse Dynamics, Intershunt, Medtronic, Merck, NIMedical, NovoNordisk, NucleusRx, NXT Biomedical, Orchestra, Pharmacosmos, PreHealth, Presidio, Procyreon, ReCor, Rockley, SCPharma, Shifamed, Splendo, Summacor, SyMap, Verily, Vironix, Viscardia, and Zoll; and receiving grants from the National Institutes of Health, Doris Duke, outside the submitted work. Robert J. Mentz has received research support and honoraria from Abbott, American Regent, Amgen, AstraZeneca, Bayer, Boehringer Ingelheim, Boston Scientific, Cytokinetics, Fast BioMedical, Gilead, Innolife, Eli Lilly, Medtronic, Medable, Merck, Novartis, Novo Nordisk, Pfizer, Pharmacosmos, Relypsa, Respicardia, Roche, Rocket Pharmaceuticals, Sanofi, Verily, Vifor, Windtree Therapeutics, and Zoll. The other authors declare no conflicts of interest.

## Supporting information


**Figure S1:** PRISMA flowchart depicting the screening and study selection process. **Figure S2:** Risk of bias assessment of included RCTs. **Figure S3:** Leave‐one‐out sensitivity; composite endpoint. **Figure S4:** Leave‐one‐out sensitivity; worsening heart failure. **Figure S5:** Subgroup analysis on the basis of drug type; composite endpoint **Figure S6:** Subgroup analysis on the basis of drug type; worsening heart failure event. **Figure S7:** Subgroup analysis on the basis of drug type; all‐cause death. **Figure S8:** Subgroup analysis on the basis of drug type; cardiovascular death. **Table S1:** Search strategy. **Table S2:** List of common variable definitions.

## Data Availability

All data generated or analyzed during this study are included in this article. Further inquiries can be directed to the corresponding author.
