# Supplementary material for: GLP‐1 and Dual GLP‐1/GIP Receptor Agonists in Heart Failure With Mildly Reduced or Preserved Ejection Fraction: A Systematic Review and Meta‐Analysis
Source: Clin Cardiol. 2025 Dec 13;48(12):e70234. doi: 10.1002/clc.70234 (PMC12701558; doi:10.1002/clc.70234)

**Supplementary Appendix**

Table S1: Search strategy

Table S2: List of common variable definitions

Figure S1: PRISMA flowchart depicting the screening and study selection process

Figure S2: Risk of bias assessment of included RCTs

Figure S3: Leave-one-out sensitivity; composite endpoint

Figure S4: Leave-one-out sensitivity; worsening heart failure

Figure S5: Subgroup analysis on the basis of drug type; composite endpoint

Figure S6: Subgroup analysis on the basis of drug type; worsening heart failure event

Figure S7: Subgroup analysis on the basis of drug type; all-cause death

Figure S8: Subgroup analysis on the basis of drug type; cardiovascular death

Table S1: Search strategy for MEDLINE (PubMed format) which was adapted for other databases.

| **Number** | **Search terms** |
| --- | --- |
| #1 | 'GLP-1 Receptor Agonists' [MeSH Terms] |
| #2 | 'Glucagon-Like Peptide 1' [MeSH Terms] |
| #3 | 'GLP-1 receptor agonist' [Title/Abstract] |
| #4 | 'liraglutide' [Title/Abstract] |
| #5 | 'semaglutide' [Title/Abstract] |
| #6 | 'dulaglutide' [Title/Abstract] |
| #7 | 'exenatide' [Title/Abstract] |
| #8 | 'lixisenatide' [Title/Abstract] |
| #9 | 'tirzepatide' [Title/Abstract] |
| #10 | #1 OR #2 OR #3 OR #4 OR #5 OR #6 OR #7 OR #8 OR #9 |
| #11 | 'Heart Failure' [MeSH Terms] |
| #12 | 'heart failure' [Title/Abstract] |
| #13 | 'cardiac failure' [Title/Abstract] |
| #14 | 'heart failure with preserved ejection fraction' [Title/Abstract] |
| #15 | 'HFpEF' [Title/Abstract] |
| #16 | #11 OR #12 OR #13 OR #14 OR #15 OR #16 |
| #17 | 'randomized controlled trial' [Title/Abstract] |
| #18 | 'Randomized Controlled Trial' [Publication Type] |
| #19 | 'RCT' [Title/Abstract] |
| #20 | #17 OR #18 OR #19 |
| #20 | #10 AND #16 AND #20 |

Table S2: List of common variable definitions

| **Study** | **Definition(s)** | |
| --- | --- | --- |
|  | Composite cardiovascular endpoint | Worsening Heart Failure Event |
| FLOW, 2024 | Composite of cardiovascular death or worsening heart failure event. The main outcomes for this analysis were the composite endpoint of time to cardiovascular death or first worsening heart failure event (defined as hospitalisation or urgent visit due to heart failure), time to first worsening heart failure event, and time to cardiovascular death | First worsening heart failure event (defined as hospitalisation or urgent visit due to heart failure) |
| SELECT, 2023 |  |  |
| STEP HFpEF, 2023 |  |  |
| STEP-HFpEF DM, 2024 |  |  |
| SUMMIT, 2024 | Adjudicated death from cardiovascular causes or a worsening heart-failure event resulting in hospitalization, intravenous drugs in an urgent care setting, or intensification of oral diuretic therapy | Adjudicated worsening heart-failure event resulting in hospitalization, intravenous drugs in an urgent care setting, or intensification of oral diuretic therapy |
| EXSCEL, 2024 | Hospitalization for heart failure or cardiovascular death | Hospitalization for heart failure |

Figure S1: PRISMA flowchart depicting the screening and study selection process

Records removed *before screening*

Duplicate records removed (n = 114)

Records identified from

PubMed/MEDLINE (n = 67)

Embase (n = 351)

Cochrane Library (n = 77)

Total (n= 495)

**Identification**

Records screened

(n = 381)

Records excluded

(n = 347)

Reports sought for retrieval

(n = 34)

**Screening**

Reports assessed for eligibility

(n = 34)

Reports excluded:

Single-arm studies, reviews, editorials, observational studies, and others (n = 28)

Studies included in meta-analysis

(n = 06)

**Included**

Figure S2: Risk of bias assessment of included RCTs


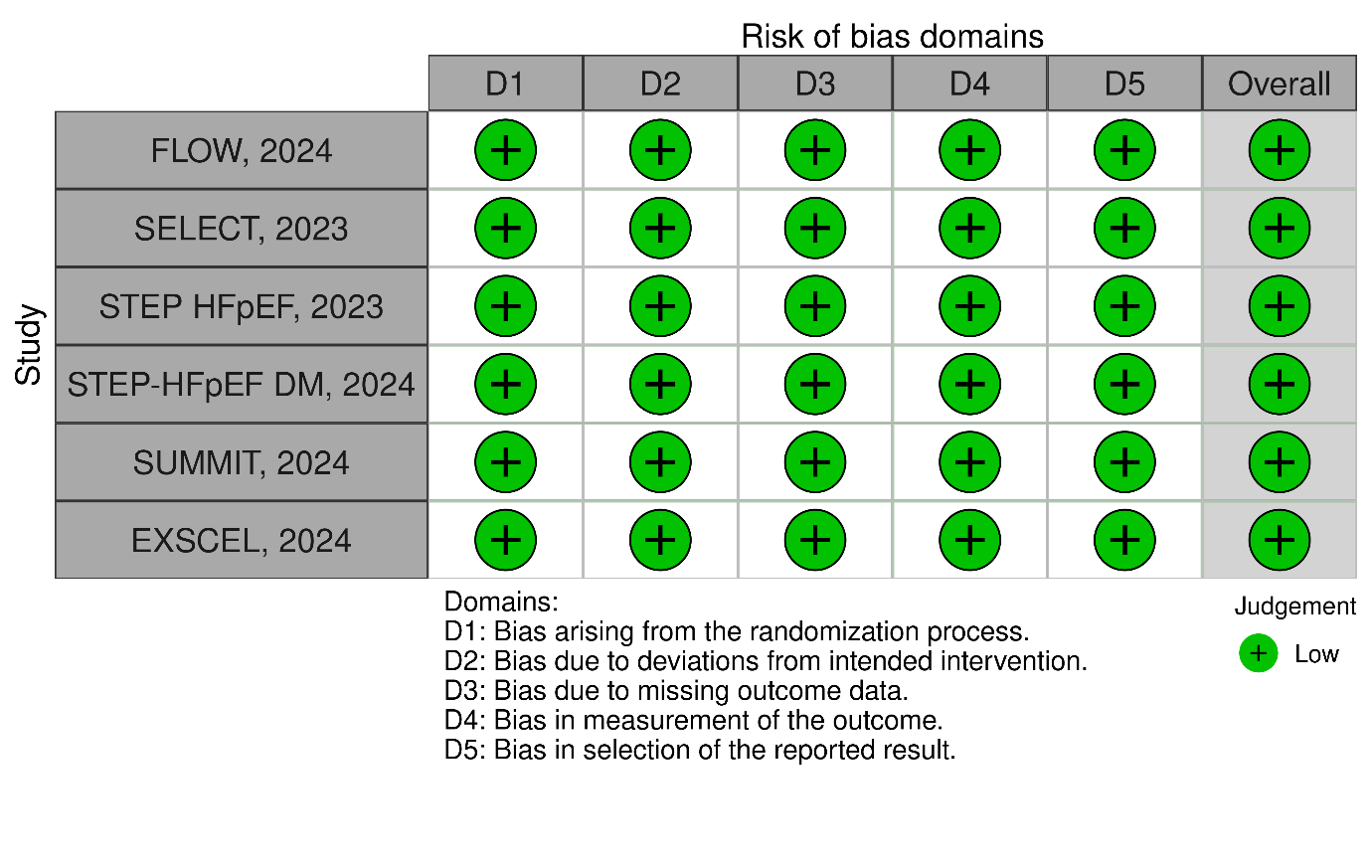


Figure S3: Leave-one-out sensitivity; composite endpoint


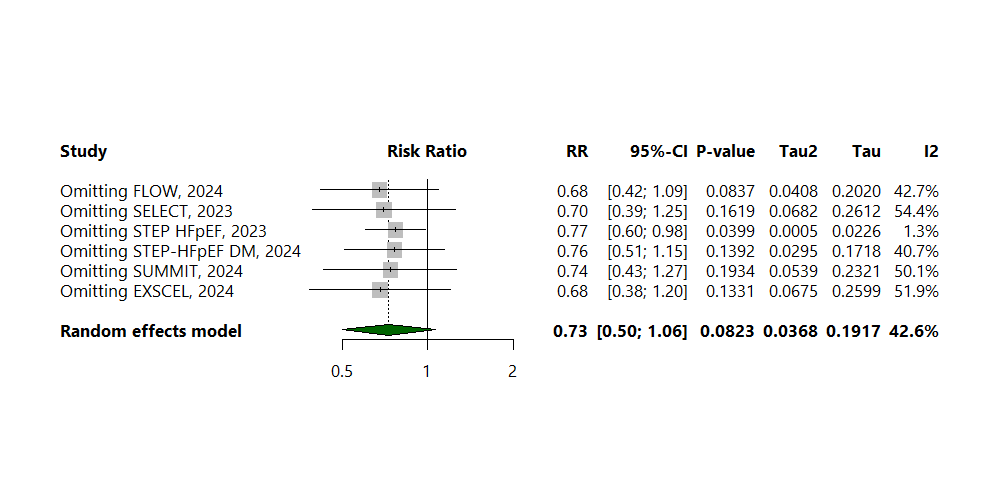


Figure S4: Leave-one-out sensitivity; worsening heart failure


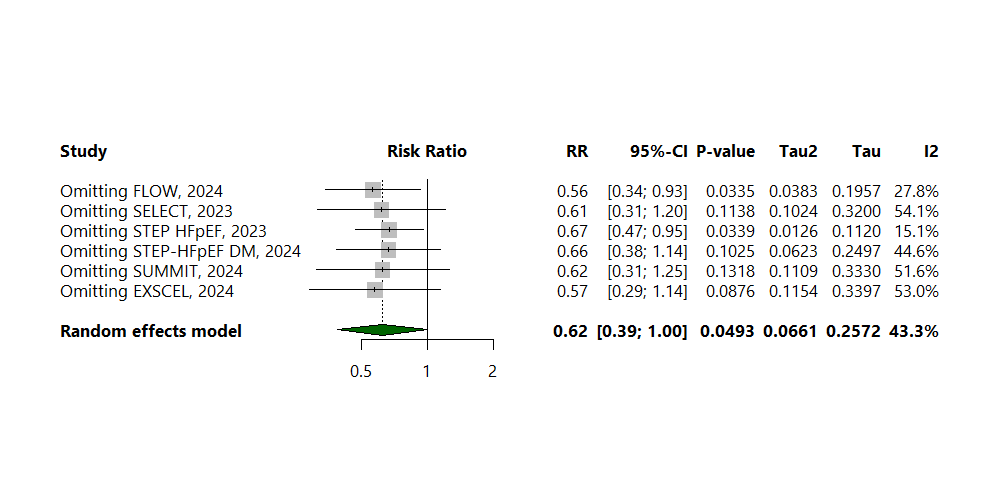


Figure S5: Subgroup analysis on the basis of drug type; composite endpoint


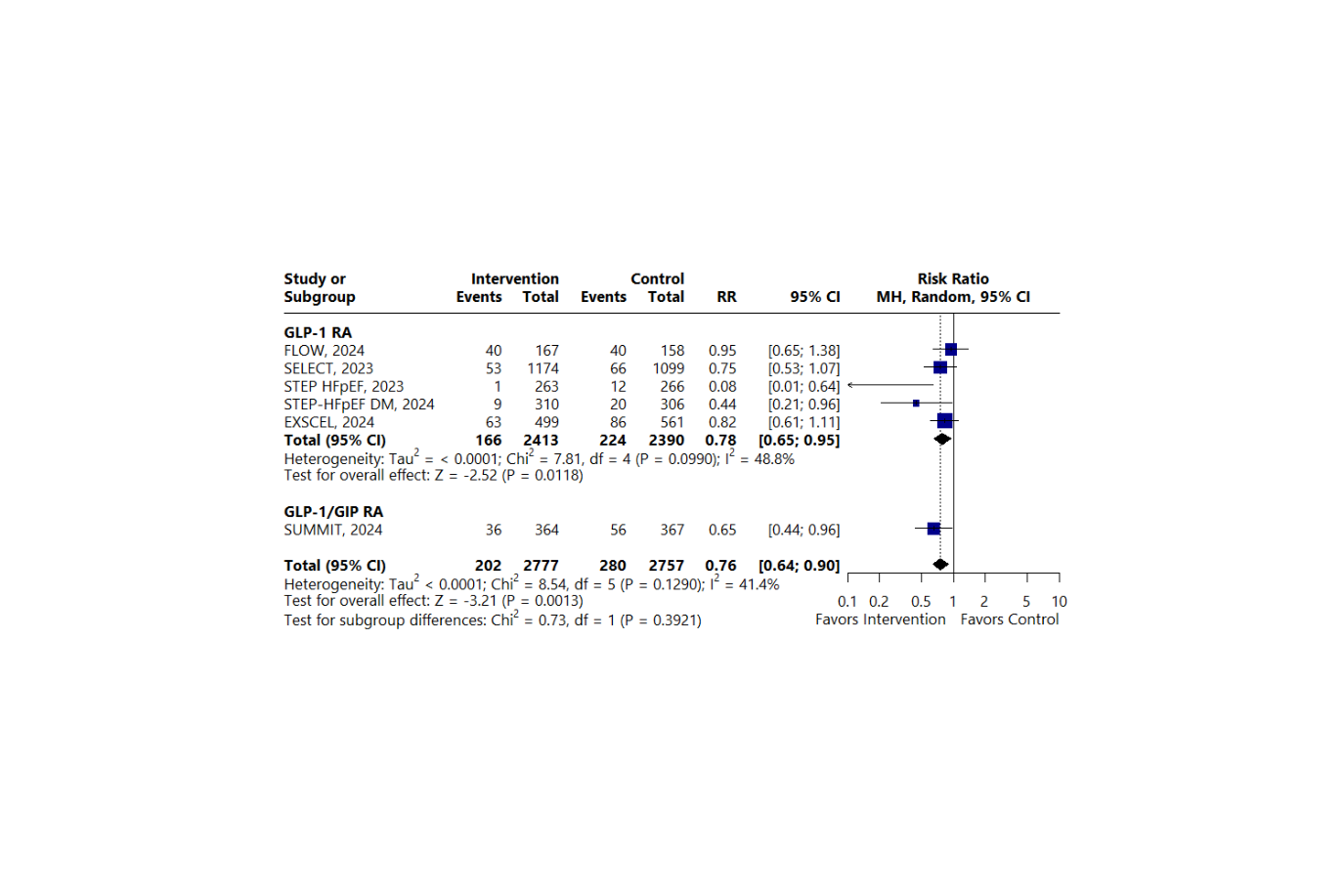


Figure S6: Subgroup analysis on the basis of drug type; worsening heart failure event


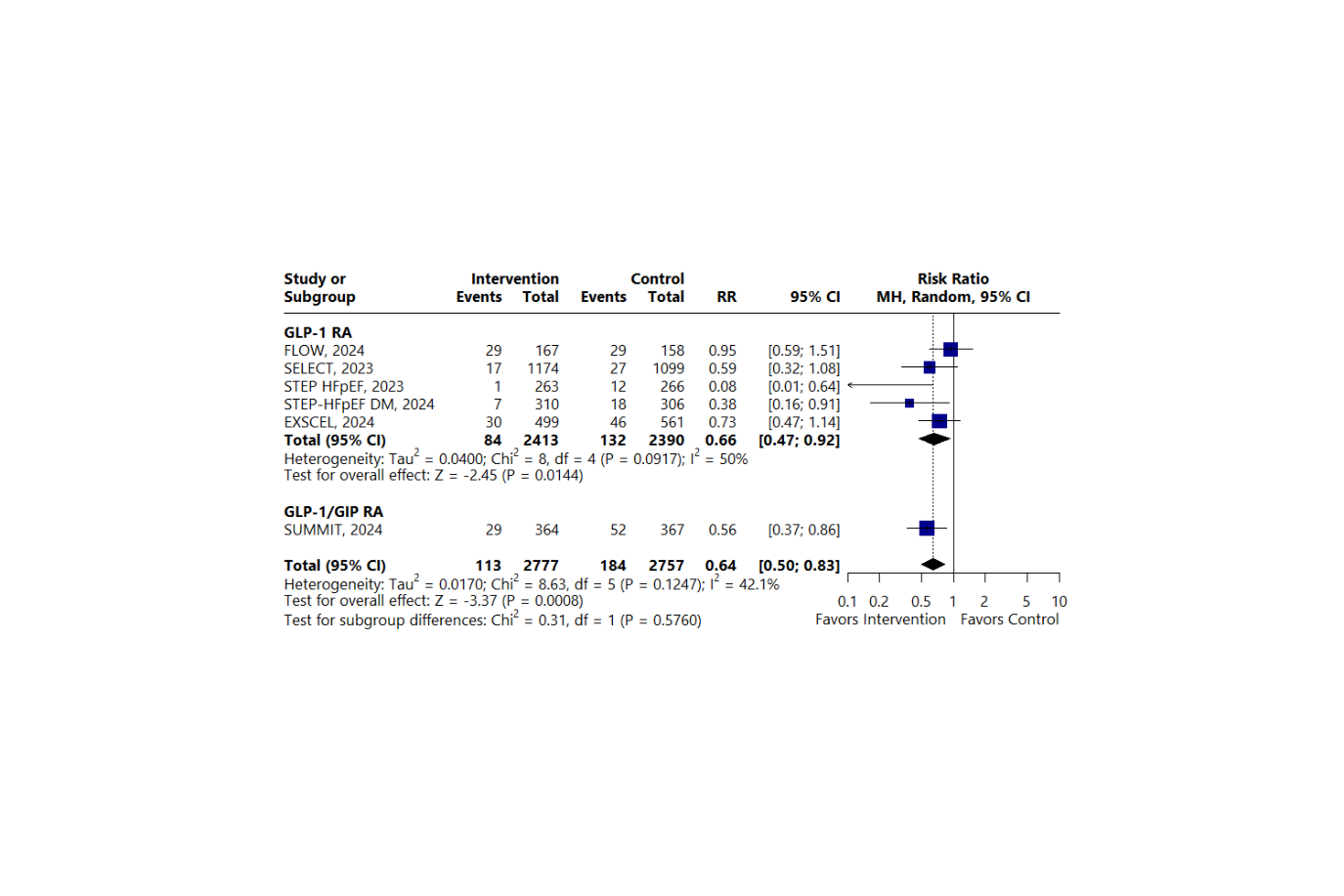


Figure S7: Subgroup analysis on the basis of drug type; all-cause death


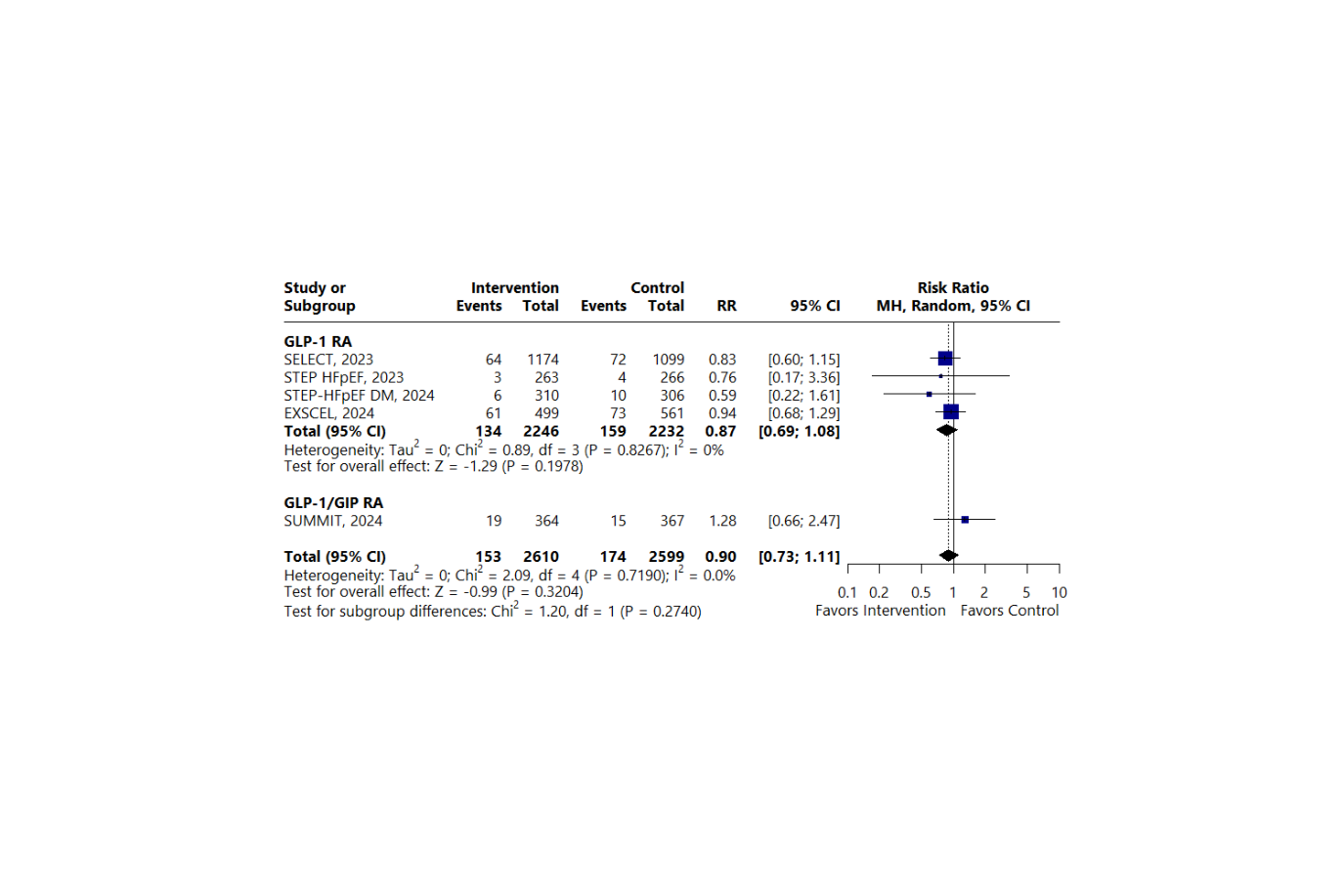


Figure S8: Subgroup analysis on the basis of drug type; cardiovascular death


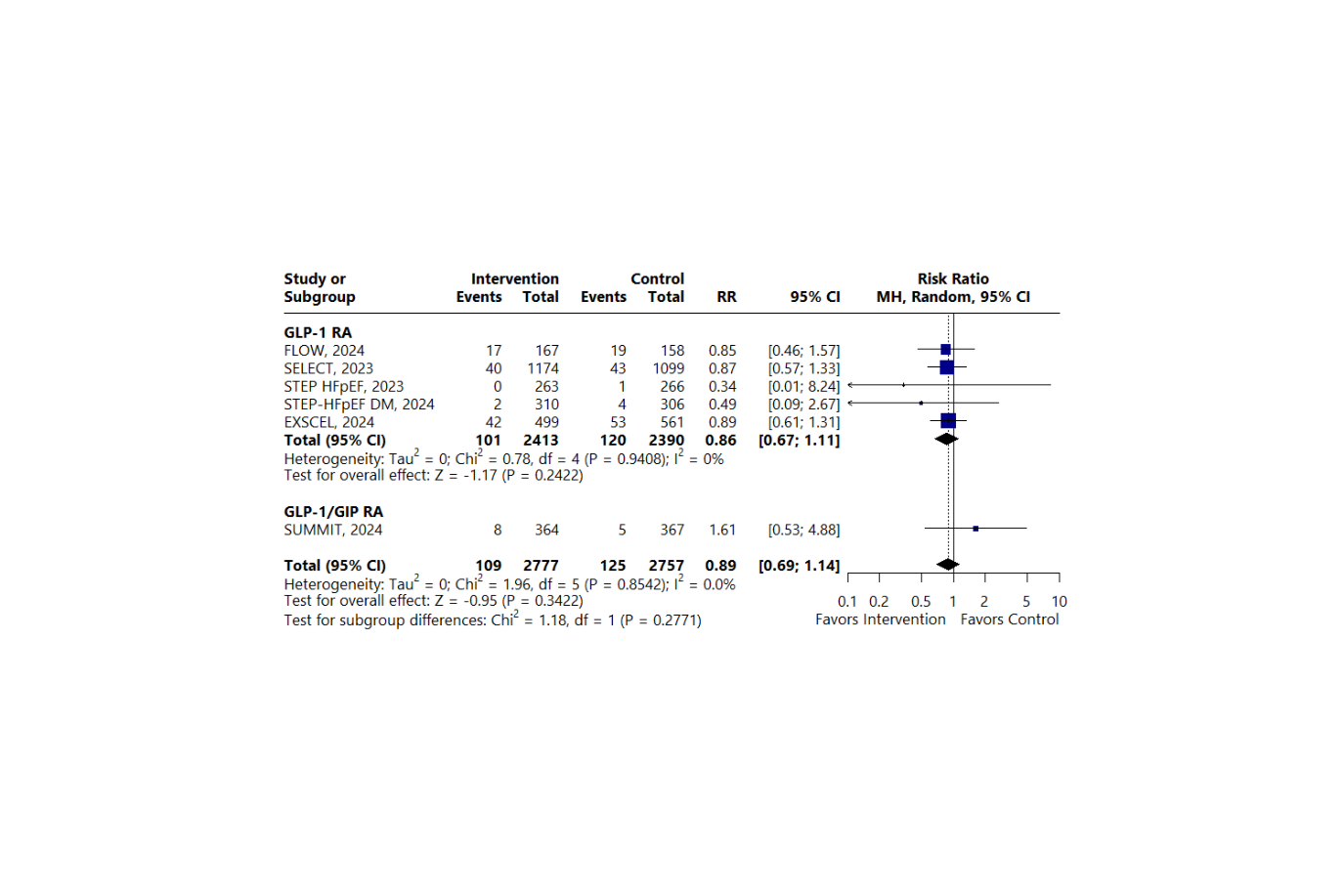

Supplement: Supplementary file 1 — Figure S1: PRISMA flowchart depicting the screening and study selection process. Figure S2: Risk of bias assessment of included RCTs. Figure S3: Leave‐one‐out sensitivity; composite endpoint. Figure S4: Leave‐one‐out sensitivity; worsening heart failure. Figure S5: Subgroup analysis on the basis of drug type; composite endpoint Figure S6: Subgroup analysis on the basis of drug type; worsening heart failure event. Figure S7: Subgroup analysis on the basis of drug type; all‐cause death. Figure S8: Subgroup analysis on the basis of drug type; cardiovascular death. Table S1: Search strategy. Table S2: List of common variable definitions. [file CLC-48-e70234-s001.docx]
